# P01-02 Can leisure time physical activity moderate the impact of occupational physical activity on sickness absence?

**DOI:** 10.1093/eurpub/ckac095.002

**Published:** 2022-08-29

**Authors:** Els Clays, Margo Ketels

**Affiliations:** Public Health and Primary Care, Ghent University, Gent, Belgium

**Keywords:** Occupational physical activity, leisure time physical activity, sickness absence, accelerometer, sustainable employment

## Abstract

**Introduction:**

Physical activity (PA) as health promotion tool is not one without adverse effects and adolescents with nonfatal physical activity-related injuries (PARI) may experience serious health consequences for the rest of their lives.

**Purpose:**

The aim of this study was to assess the associations between physical activity-related injuries in adolescents in various settings and moderate-to-vigorous physical activity, medically attended injuries, cardiorespiratory fitness and body composition.

**Methods:**

As a part of the pilot study of the Health Behaviour in School-aged Children Study conducted in October and November 2021 in Slovakia, we surveyed 119 adolescents (53 girls; average age 12,6±2,0) for frequency ofmoderate-to-vigorous physical activity (MVPA), frequency of medically attended injuries (MAI), frequencies of physical activity-related injuries in sports clubs (PARISC), physical activity-related injuries in leisure-time (PARILT) and physical activity-related injuries in schools (PARIS) and we measured their cardiorespiratory fitness (using 20-metre shuttle run) and their body composition (using InBody 230).

**Results:**

Out of 119 adolescents, 50 (42%) were attending sports clubs of which 27 (54%) had one or more PARI in sports clubs' activities in previous year, 50 adolescents (42%) had PARI in leisure activities and 15 (12,6%) in school activities. PARISC led to an average of 10 missed days from school or leisure-time activities. PARILT led to 7,2 missed days and PARIS led to 6,2 missed days. Spearman's correlations (n = 50 for PARISC and n = 119 for PARILT and PARIS) revealed associations between MAI and PARISC, PARILT and PARIS, but not between MVPA or 20-metre shuttle run laps and PARISC, PARILT and PARIS. Not surprisingly, percentage of body fat was negatively associated with the number of 20-metre shuttle run laps. In addition, results of crude linear regression models showed that frequency of MVPA was not associated with frequencies of PARISC (B coefficients (B)/95% CI: 0,03/-0,11-0,18), PARILT (B/95% CI: 0,04/-0,05-0,13) or PARIS (B/95% CI: -0,02/-0,07-0,02) among Slovak adolescents in our pilot study.

**Conclusions:**

Estimating the burden of PARI is important in advocating the need of directing sufficient resources to PARI prevention along with the PA promotion. Improvement and understanding of factors associated with PARI might be helpful in PARI prevention. In addition, it might, among other factors, play a role in promotion of active lifestyle in adolescence.

